# Serum Cytokinome Profile Evaluation: A Tool to Define New Diagnostic and Prognostic Markers of Cancer Using Multiplexed Bead-Based Immunoassays

**DOI:** 10.1155/2016/3064643

**Published:** 2016-12-06

**Authors:** Francesca Capone, Eliana Guerriero, Angela Sorice, Giovanni Colonna, Gennaro Ciliberto, Susan Costantini

**Affiliations:** ^1^CROM, Istituto Nazionale Tumori “Fondazione G. Pascale”, IRCCS, Naples, Italy; ^2^Center of Medical Informatics, SIM/AOU, Second University of Naples, Naples, Italy; ^3^Direzione Scientifica, Istituto Nazionale Tumori “Fondazione G. Pascale”, IRCCS, Naples, Italy

## Abstract

In recent years, many researchers are focusing their attention on the link between inflammation and cancer. The inflammation is involved in the tumor development and suppression, by stimulating the immune response. In particular, the transition from chronic inflammation to cancer produces angiogenic and growth factors able to repair the tissue and to promote cancer cell survival, implantation, and growth. In this contest, the cytokines contribute to the development of these processes becoming active before and during the inflammatory process and playing an important function at the various disease levels. Thus, these proteins can represent specific markers of tumor development and progression. Therefore the “cytokinome” term is used to indicate the evaluation of cytokine pattern by using an “omics” approach. Newly, specific protein chips of considerable and improved sensitivity are being developed to determine simultaneously several and different cytokines. This can be achieved by a multiplex technology that, through the use of small amounts of serum or other fluids, is used to determine the presence and the levels of underrepresented cytokines. Since this method is an accurate, sensitive, and reproducible cytokine assay, it is already used in many different studies. Thus, this review focuses on the more latest aspects related to cytokinome profile evaluation in different cancers.

## 1. Introduction

The most accepted model of the onset of cancer in humans shows that often, but not always, one or more genes that encode components of major signaling pathways are specifically altered in a single individual and this is the starting step driving the tumor. This event is typically repeated; thus we can have sequential mutations of these pathways along the individual's life at the transition between each tumor stage. In the final stage we often see the spread of cancer through its cancerous metastases. However, the routinely massive sequencing of genes involved in cancer has unequivocally shown that in the most common forms of human cancer we can observe that only few genes are altered in a high percentage of cancers, while the great majority of them is altered infrequently [1]. Therefore, nevertheless many scientific articles massively support the paradigm that cancer originates from mutations in specific genes, on the basis of existing data; the mechanisms for cancer diffusion through metastases must be broader than is typically thought. For example, genomic analyses of three subtypes of brain tumor show that one subtype carries a chromosomal translocation generating a new tumor-driving gene, while another subtype lacks tumor-driving mutations but possesses anomalous epigenetic modifications, and, finally, the third subtype shows neither gene mutations nor epigenetic modifications [2–4]. These observations (and others too) are confusing due to the fact that the basic biology of cancer is still a black box; evidently more complex systemic processes are involved. Therefore, the failure of numerous drugs and treatment have only achieved increased survival but not a cure for cancer.

Over the past several years, there is accumulating evidence that chronic inflammation is involved in the development and progression of cancer [5]. The cancer cells are able to spread around a cell-to-cell information by means of a broad family of small inflammatory signaling proteins, that is, cytokines, which favor tumor growth, both facilitating genomic instability and stimulating the angiogenesis. Cytokines include chemokines, interferons, interleukins, lymphokines, and tumor necrosis factor. Cytokines are secreted by numerous cell types, including immune cells like macrophages, B lymphocytes, T lymphocytes, and mast cells, but also by endothelial cells, fibroblasts, and various stromal cells. In general, a specific cytokine may be secreted by more than one type of cell [5].

For example, macrophages are innate immune cells contributing to tumor growth and progression by their trophic role that facilitates angiogenesis, matrix breakdown, and tumor-cell motility and by promoting chronic inflammation [6]. Macrophages produce inflammatory cytokines such as TNF, IL-6, IL-12b, and IL-23 that drive inflammation through TLR signaling [7]. However, TLR signaling is insufficient to explain the strong activation of inflammatory cytokine genes in human macrophages, as previously proposed [8]. Rather, it has been found that the synergistic activation of these genes acts through interferon-*γ* (IFN-*γ*) and Toll-like receptor (TLR) signaling as important mediators of innate immunity and inflammatory disease pathogenesis [9], where IFN-*γ* primes macrophages for synergistic transcription of inflammatory cytokine genes upon stimulation with inflammatory factors such as TLR ligands [10]. Indeed, genome-wide analysis has shown priming of regulatory elements by IFN-*γ* with a synergistic induction of the transcription, so as to provide a proper chromatin environment to augment TLR-induced gene transcription. This provides transcriptional responses able to remodel the chromatin as well as support inhibition of Jak-STAT1 signaling [10].

Indeed, the inflammatory component of a developing neoplasm often includes a very differentiated leukocyte population such as neutrophils, dendritic cells, macrophages, eosinophils, and mast cells, as well as lymphocytes able to produce an assorted array of cytokines, chemotactic cytokines (or chemokines), and soluble mediators of cell killing, such as TNF-*α*, interleukins, growth factors, and interferons (IFNs) [11, 12]. Instead, the evolutionarily well conserved inflammatory response of higher organisms is regulated by cytokines, which are released by cells and affect the behavior of other cells, by exerting pleiotropic and redundant effects on growth promotion, differentiation, and activation, in normal cells as well as in chronic diseases such as cancer [11, 12].

Recently Mlecnik et al. (2016) proposed an immunoscore that could be used as a immunological biomarker to predict metastasis guiding the therapy [13]. However, since the genetic alterations of tumor cells did not show any relationship with the fact that the tumor develops metastasis, this should suggest that the change is a cause, rather than a consequence, of metastasis. Hence, the production of cytokines by tumor cells seems to be the molecular perturbation responsible for the development of malignant tumors [14], and this underscores the role of tumor derived cytokines.

## 2. Cytokinome Definition

Cytokine production and control are highly complex and multifactorial, and their effects are reflected through multiple regulatory subnetworks [11, 12]. Indeed, the cellular response to stimuli requires a perfect coordination between cellular receptors and intracellular metabolic network to integrate external stimuli and activate effective metabolic responses. The effective cellular response is in turn mediated through cytokines [11, 12]. These small informational molecules have a flow that is essentially external to the cell, covering the cell-to-cell communications; therefore, the cytokines as such do not fall in the metabolic networks that are intracellular. Their communication takes place via asynchronous modes, which is safer and energetically more economic for a multicellular system, but also independent of space and time constraints [15]. Actually, asynchronous mode means that the cell (sender) sends informational molecules without waiting for the response, but the message (cytokine) goes through a fairly homogeneous medium up to a waiting receiver, which is the receptor of another cell of the same type or even different. The receptor, which is an intermediate, receives, authenticates, and pours the message towards the inside by means of specific signaling pathways, in order to reach the true receiver which is normally the cell nucleus.

This mode, seemingly very complex, has along the way several filters that authenticate the message itself, making it sure. Furthermore, the pleiotropy ensures that the message arrives anyway. This complexity suggests that only an integral and simultaneous knowledge of the role of different cytokines may help to describe their role in the pathogenesis of cancer instead of a single cytokine assessment [16].

The “omics sciences” (genomics, transcriptomics, proteomics, fluxomics, metabolomics, etc.) are actually used to study the living organisms as a whole system. The cytokinome, according to the “omics” system of definition, can be defined as the set of all cytokines, inclusive of their mechanisms of interaction with and around cells [17]. The complexity of the cytokine system in humans can be more clearly elucidated using the cytokinome, which evaluates the complex network of interactions used to regulate either cytokine synthesis or their cognate receptors [18].

**Figure 1:**
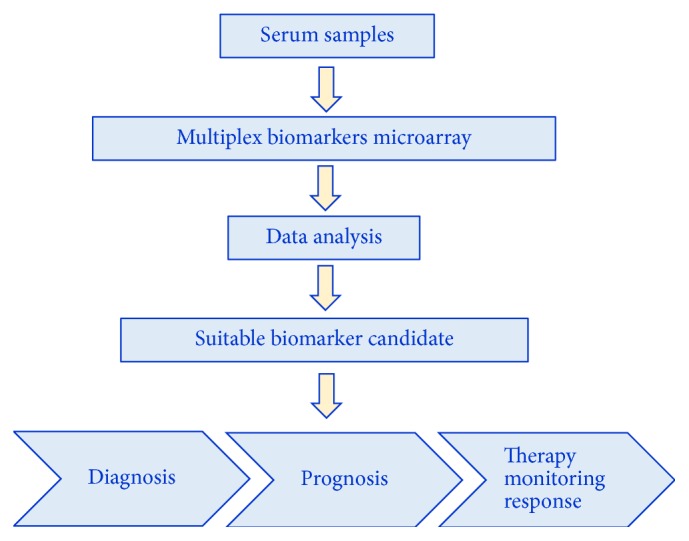
Flowchart related to a broad-spectrum bead-based multiplex immunoassay.

In fact, the cytokinome goes deeper in studying antagonistic and synergistic interactions among different cytokines, which can occur in many different and often redundant ways [19]. Therefore, a comprehensive understanding of cytokine functions, as well as a correct knowledge of their functional role, can be obtained only through simultaneous and coherent measurements of the cytokines concentrations in serum. This point raises the inherent difficulty of a simultaneous measurement of the cytokine concentrations to obtain correct internal ratios among the various molecules present in the same biological fluid and also due to the often large difference in concentrations spanning several magnitude orders. At present, it is possible to effectively characterize the serum levels of cytokines using multiplexed bead-based immunoassays [18] (Figure 1).

This assay is based on the evaluation of large panels of cytokines in patient sera. The related concentrations are analyzed to identify new biomarker candidate(s), which can be used to improve the cancer diagnosis or prognosis and to evaluate the treatment response monitoring them over time.

In this review we comment on studies in which the cytokinome profile has been evaluated on sera from patients affected by several cancer types (Table 1). In this way we can evidence the importance of this approach as a tool to analyze the interaction network of cytokines both in healthy individuals and in patients affected by different cancers.

## 3. “Cytokinome” and Breast Cancer

Considerable advances have been made in recent years in understanding the genetics and molecular biology of breast cancer, but, for women in most western countries, this cancer remains still a major cause of death [20]. It is a heterogeneous disease and is divided into 4 main subtypes according to its clinical molecular characteristics as luminal A and luminal B and HER2 amplified and triple negative tumors. Each subtype harbors specific clinical behavior and aggressiveness, which affect disease prognosis. Luminal tumors present positivity to estrogen and/or progesterone receptors (luminal A) and can further present the amplification of the receptor of the human epidermal growth factor 2 (HER2). The latter is categorized as luminal B and is more aggressive than luminal A cancers. Some tumors present only the overexpression of HER2 and are named as HER2-amplified. Recently we have demonstrated that the positive and negative estrogen tumors show different metabolic patterns (*manuscript in press*). Finally, tumors that do not exhibit any of these receptors are classified as triple negative [21]. Breast cancer develops within a specialized tumor microenvironment that consists of numerous cell types including cancer cells, stromal cells, adipose, and infiltrating immune cells. These cells release a wide range of factors that can modulate tumor development by regulating cancer cell proliferation, survival, invasion, motility, and angiogenesis [22]. In this cancer the inflammation has an important role in tumor initiation, promotion, angiogenesis, and metastasis, and, hence, the cytokines are prominent players [23].

Depending on the staging of disease, women with breast cancer exhibit distinct patterns of circulating cytokines compared to healthy control. In the early stages, when breast cancer is localized, the patients display reduced serum levels of TNF-*α* and IL-12. On the other hand, patients presenting advanced disease have high systemic levels of TNF-*α* and IL-1*β*. Therefore, the cytokine profile is closely related to tumor subtype and may affect disease outcome in some instance [24]. Nicolini et al. (2006) showed that in breast cancer some cytokines such as IL-1, IL-6, IL-11, and TGF-*β* stimulate breast cancer proliferation and/or invasion while others such as IL-12, IL-18, and IFNs inhibit it. In particular, IFN-*β* has been reported to enhance estrogen and progesterone receptors [25]. Similarly, high circulating levels of some cytokines seem to be favorable prognostic indicators such as soluble IL-2R while others are unfavorable, such as IL-1b, IL-6, IL-8, IL-10, IL-18, and gp130. However, IL-2 is a potent stimulator of cellular immunity and, for this property, is the most selected interleukin for clinical trials [26]. Overall, these data underline the important role of cytokinome profile by which a global approach on assessing multiple cytokine concentrations as a measure of the interaction between the immune system and the tumor may potentially yield new methods for the diagnosis and/or prognosis of cancer patients [27].

## 4. “Cytokinome” and Ovarian Cancer

The ovarian cancer is often asymptomatic and its clinical diagnosis is made when the disease is advanced and has already created metastases; for these reasons it represents the third most common gynecologic malignancy in the woman [28, 29]. Ovarian cancer has a distinctive biology and behavior at the clinical, cellular, and molecular levels and its early detection might improve clinical outcome. Inflammation is a common feature of ovarian cancer, and measurement of plasma markers of inflammation is useful for identifying candidate markers for use in screening evaluation of patients with cancer [28–30]. In serum of patients with ovarian cancer different cytokines with diagnostic value involved in the different cancer aspects were evaluated.

Many angiogenic factors are expressed and produced by tumor cells, such as platelet-derived growth factor (PDGF), basic fibroblast growth factor (bFGF), VEGF, interleukins such as IL-6, IL-8, IL-1*α*, and IL-1*β*, and other cytokines, such as MCP-1, granulocyte CSF (G-CSF), M-CSF, and tumor necrosis factor-*α* (TNF-*α*). Tumor-released cytokines and their receptors expressed by endothelial and hematopoietic/lymphoid cells can induce the increase of the production also of other types of cytokines [31]. Other studies demonstrated the importance of identifying additional plasma-based proteins to predict the disease and significantly improving prediagnostic decision-making in patients with ovarian cancer, particularly the IL-6 secretion that occurs early in the pathogenesis of this cancer [52]. Also in this cancer the cytokinome profiling is of high diagnostic power and valuable tool to identify multiple soluble factors useful for its early diagnosis and therapy response assessment [32]. In particular, Matte et al. (2012) applied a multiplex cytokine array technology to measure the level of 120 cytokines in ascites from 10 ovarian cancer patients [32]. These authors demonstrated that the levels of several factors including, among others, angiogenin, angiopoietin-2, GRO, ICAM-1, IL-6, IL-6R, IL-8, IL-10, leptin, MCP-1, MIF NAP-2, osteoprotegerin (OPG), RANTES, TIMP-2, and UPAR were elevated in most malignant ascites and that IL-10 promoted the antiapoptotic activity of malignant ascites. In fact, the levels of IL-10 below 24 pg/mL in ascites correlated with survival on patients with ovarian cancer. Therefore, a better understanding of the cytokine network is essential to determine the role of ascites in ovarian cancer progression and could be more indicative of the tumor environment [32].

## 5. “Cytokinome” and Lung Cancer

Lung cancer results in the largest number of cancer-related deaths worldwide. More than 85% of those cases are currently classified as non-small-cell lung cancer (NSCLC), for which the predicted 5-year survival rate is 15.9% [53]. The predominant risk factor for lung cancer is smoking, accounting for approximately 90% of these lung cancer deaths. Additionally, lung cancer risk is associated with several indicators of inflammation, including pulmonary fibrosis, chronic obstructive pulmonary disease, and chronic pulmonary infections [53]. Since inflammation process is a complex response to stimuli involving the interplay of host cells and signaling molecules, such as proinflammatory and anti-inflammatory cytokines, growth and angiogenesis factors, and chemokines, the lung cancer risk has been associated with higher levels of sTNFRII, IL-7, TGF-*α*, CXCL5, CXCL9, CXCL13, CCL17, and CCL22 [33]. Recent evidence has shown that circulating inflammatory cytokines are associated with survival in early-stage lung cancer, and CCL15 was significantly associated with poor survival [34]. Pine et al. (2011) have focused their studies on the association between C-reactive protein (CRP), IL-6, and IL-8 and lung cancer risk [35]. Moreover, Barrera et al. (2015) showed that in patients with NSCLC there is a complex network existing between inflammatory, anti-inflammatory, angiogenic, and antiangiogenic cytokines. In particular, higher levels of IL-6, IL-8, IL-12, IL-17, and IFN-*γ* and lower levels of IL-33 and IL-29 are in NSCLC patients compared to healthy controls [36]. Also in lung cancer, the cytokinome profile has highlighted the opportunity to comprehensively evaluate the role of a large number of circulating inflammatory markers representative of the assortment of immune-related processes and pathways in cancer etiology and prognosis [54]. In fact, when the levels of many markers using Bio-Rad and Millipore kits were evaluated on a great number of cancer-free participants, it was possible to underline that a majority of markers were detectable in >25% of individuals on all specimen types/kits [54]. In particular, 45 Bio-Rad and 71 Millipore markers had acceptable performance and the median concentrations and intraclass correlation coefficients (ICCs) differed to a small extent across specimen types and to a large extent between Bio-Rad and Millipore [54]. Therefore, the use of this technology is reliable to perform patient serum screening.

## 6. “Cytokinome” and Colorectal Cancer

Colorectal cancer (CRC) is a leading cause of cancer death in developed countries [55] and cancer-related inflammation appears to be a hallmark of CRC as evidenced by the increased risk of CRC in the setting of chronic bowel inflammation [38]. Accumulating evidence suggests that some cytokines and their receptors act as regulator keys of the tumor microenvironment and are involved in many pathological entities ranging from inflammatory bowel disease to carcinogenic processes, such as the autonomous growth signaling, which influences tumor growth, invasion, and metastasis [56]. Studies have shown that CRC development is accompanied by alterations in cytokine production, which is thought to polarize from Th1 into Th2 along the colorectal adenoma-carcinoma sequence. Increased serum cytokine concentrations of IL-6, IL-7, IL-8, and PDGF, and lower levels of MCP-1 have also been reported in CRC patients compared with healthy individuals. Moreover, advanced CRCs were associated with higher levels of IL-8, IL-1ra, and IL-6 [37]. However, some other cytokines and their receptors have been found to promote tumor growth, invasion, and metastasis by acting as key regulators of the tumor microenvironment and by being involved in many carcinogenesis-related processes, such as inflammation and autonomous growth signaling. It is therefore clinically important to elucidate the influences of multiple cytokines on the prognosis of colorectal cancer patients. For example, a recent report showed a novel cytokine-based prognostic classifier (CBPC) for prognostic prediction where 17 different circulating cytokines in metastatic CRC, such as FGF-2, TGF-*α*, Flt-3L, GM-CSF, INFa2, GRO, IL-10, MCP-3, MDC, sIL-2Ra, IL-2, IL-7, IL-8, MCP-1, MIP-1*β*, TNF-*α*, and VEGF, resulted in being able to predict metastatic CRC patients with a high risk of short OS [57]. Also in this cancer it has been determined that a cytokinome profile provides a simultaneous evaluation of multiple biomarkers in the same sample, and, by examining changes in multiple cytokines, it may be possible to detect more specific “cytokine footprints” for different inflammatory and neoplastic diseases. Therefore, an analysis of extensive sets of cytokines would provide more accurate information on the tumor-related immunological responses, thus bringing out the importance of individual cytokines on the immune response against cancer [58].

## 7. “Cytokinome” and Liver Carcinoma

Many groups are focusing their attention on the hepatocellular carcinoma (HCC) because 700,000 cases are diagnosed in each year [59]. Different factors can induce its development such as alcoholic liver disease, chronic infection with hepatitis B and hepatitis C virus, intake of aflatoxins-contaminated food, obesity, and type 2 diabetes (T2D) [60]. However, often the patients develop firstly liver fibrosis and cirrhosis and, then, HCC [61]. Our group has recently evaluated a large panel of cytokines in patients subdivided in four groups, T2D, HCV, HCV-related HCC alone, and HCV-related HCC with T2D, compared to healthy controls, to identify new markers specific for diabetes and/or HCC [39]. The obtained data evidenced that the levels of HGF, leptin, sVEGFR-1, sVEGFR-2, IL-2R, s-IL-6Ra, and IL-18, were higher in patients with T2D, HCV, and HCC. On the other hand, the levels of *β*-NGF, CXCL1, CXCL9, CXCL12, IL-16, and PECAM-1 increased in patients with HCV and HCC and those of IFN-*α* and Prolactin only in HCC patients [39].

Searching markers specific for the copresence of T2D and HCC, our results demonstrated the level increase of three interleukins (IL-2R, IL-16, and IL-18), sIL-6Ra, two chemokines (CXCL1 and CXCL12), ADIPOQ, *β*-NGF, HGF, and IFN-*α* and the decrease of leptin in T2D-HCC patients compared to those with only T2D or HCC. Moreover the comparison between the cytokine levels in T2D and T2D-HCC patients showed that CXCL9, PECAM-1, Prolactin, and glucagon levels were higher in T2D-HCC patients compared to T2D patients, and VEGFR-1 and sVEGFR-2 were lower in T2D-HCC patients [39]. Hence, also in the case of multifactorial HCC, the cytokinome analysis can be used to identify new markers useful for improving the prognosis of its progression [39].

## 8. “Cytokinome” and Melanoma

Melanoma is a malignant disease about which it is important to underline that if it is diagnosed in early stages, the disease is highly curable but if it is diagnosed at an advanced metastatic stage, its prognosis becomes poor [62]. However, the melanoma is an heterogeneous disease, and its pathogenesis depends on DNA mutations leading to a malignant transformation that induces an increased production of multiple growth factors and cytokines [63]. Different studies have highlighted the cytokines role in melanoma. In particular, the paper of Yurkovetsky et al. (2007) is considered the first broad multimarker screening of serum proteins for cytokines and other proinflammatory and proangiogenic proteins of patients with melanoma. This screening has shown that concentrations of 15 biomarker proteins (IL-1*α*, IL-1*β*, IL-6, IL-8, IL-12p40, IL-13, G-CSF, MCP-1, MIP-1*α*, MIP-1*β*, IFN-*α*, TNF-*α*, EGF, VEGF, and TNF-RII) were significantly higher in patients with resected high-risk melanoma compared with healthy controls. Moreover, IFN-alpha2*β* therapy resulted in a significant decrease of serum levels of immunosuppressive and tumor angiogenic/growth stimulatory factors (VEGF, EGF, and HGF) and increased levels of IP-10 and IFN-*α* [40]. Moreover, Shetty et al. (2013) evidenced that the levels of MIP-1*α*, IL-1R*α*, IL-1*β*, IL-1*α*, IL-17, EGF, IL-12p40, VEGF, GM-CSF, and MIP-1*β* were significantly higher in normal controls compared to melanoma patients, while IP-10 level was lower [64].

## 9. “Cytokinome” and Gastric Cancer 

Chronic inflammation of the gastric epithelium contributes to the pathogenesis of gastric cancer (gastric cancer) [65]. This includes a sequence of events that often begins with* Helicobacter pylori*-induced chronic superficial gastritis, progressing towards atrophic gastritis, intestinal metaplasia, dysplasia, and eventually GC [65]. Indeed* Helicobacter pylori* is etiologically involved in the development of gastric cancer and infected gastric mucosa has been shown to possess elevated levels of cytokines, for example, interleukin (IL-1*β*, IL-6, and IL-8) [41]. Another study reported an increase in gastric cancer risk with increased level of some cytokines (IL-1*β*, IL-2, IL-4, IL-6, IL-8, IL-10, TNF-*α*, and IFN-*γ*) and evidenced that, in a population with high gastric cancer incidence and high* H. pylori* prevalence, increased circulating levels of IL-8 may indicate increased risk of gastric cancer [42]. Also our group has evaluated a large panel of cytokines characteristic of patients with gastric cancer comparing them with healthy controls. Higher amount of IL-8, eotaxin, HGF, IP-10, MIP-1b, VEGF, Gro-a, IL-2R, IL-18, M-CSF, MIF, and MIG was secreted by adenocarcinoma gastric cancer (AGC) with respect to control group [*manuscript in preparation*].

## 10. “Cytokinome” and Pancreatic Cancer

Pancreatic cancer is the fifth-most-common cause of cancer and its incidence is increasing at an alarming rate in western countries [66]. Unfortunately, the molecular mechanisms underlying the development and progression of the pancreatic cancer have not yet been clarified [66]. In 1999 the first paper that reported an increase in IL-10 and TGF-*β* concentrations in the sera of pancreatic cancer patients and an increase in IL-4 production and a decrease in IFN-*γ* and IL-12 production from stimulated PBMCs was published, thus demonstrating a skewing of T-cell cytokine production towards a Th2 [43]. By multiplexing profiles of cytokines, Gabitass et al. (2011) confirmed this Th2 skewing in pancreatic cancer and extended these observations to esophageal and gastric cancer, demonstrating statistically significant increases in the plasma concentrations of the Th2 cytokines IL-5, IL-6, and IL-10 in patients when compared with controls [44]. Finally a recent study has shown higher levels of IL-6, IL-8, IL-10, and TNF-*α* and lower levels of IL-23 in pancreatic cancer patients compared to healthy controls [45]. This study has evidenced that these cytokines may support the blood vessels formation in the tumor microenvironment, contributing to metastatic spread by influence on the immune cells activity, inflammatory processes intensity, and the tumor cells invasiveness [45].

## 11. “Cytokinome” and Renal Cancer

Renal cell carcinoma (RCC) accounts for approximately 3% of adult malignancies and 90–95% of neoplasms arising from the kidney [67]. This disease is characterized by a lack of early warning signs, diverse clinical manifestations, and resistance to radiation and chemotherapy. Approximately 30% of patients with renal carcinoma (RCC) present with metastatic disease [67]. Negrier et al. (2004) evaluated the levels of IL-6, IL-10, and VEGF in patients with metastatic RCC. The results of this study showed that (i) none of these cytokines was associated with response to treatment, (ii) IL-10 was unrelated to progression-free or overall survival, and (iii) IL-6 was statistically significant in relation to progression-free survival and overall survival [46]. Moreover, a large panel of cytokines and angiogenic factors was evaluated for study if there was a correlation between the serum cytokine levels and overall survival in patients with metastatic renal cell carcinoma treated with interferon-alpha (IFN-*α*). This study demonstrated that serum levels of IL-5, IL-12, IL-6, and VEGFA contributed to prognostic evaluation in mRCC and to identify patients with 20% 5-year OS [47]. Also in our group we evaluated the serum levels of cytokines and chemokines in RCC patients to identify a phenotype that could be informative and prognostic in these patients. We evidenced an increase of the levels of IL-6, IL-8, IL-10, G-CSF, CXCL10, CXCL11, HGF, and VEGF in the serum of RCC patients compared to healthy controls by using a consensus of three different multiplex biometric ELISA-based immunoassays [48]. Recently, Pal et al. (2015) have assessed plasma levels of cytokines and growth factors in patients with metastatic renal cell carcinoma treated with pazopanib and subdivided into responders and nonresponders. In particular, the authors have not found significant differences between the two groups of patients in baseline whereas at 6 months of follow-up the nonresponders ended up having higher levels of HGF, IL-2R, IL-6, and IL-8, and VEGF compared to responders. This suggested that the drug resistance mechanism is at least partially related to the systemic tumor immune tolerance generation [68].

## 12. “Cytokinome” and Thyroid Cancer

Thyroid cancer is an uncommon type of cancer and it is usually diagnosed early. However, after the treatment this cancer may come back, also many years after treatment [69]. The most common malignant thyroid neoplasms are the well-differentiated papillary thyroid carcinomas, the follicular thyroid carcinoma, and the undifferentiated anaplastic carcinomas [69]. Data from clinical trials have shown that only few and limited positive responses were obtained with chemotherapeutic drugs in thyroid cancer. Unfortunately, the molecular bases involved in the chemotherapy-based regimens failure have not been established in most thyroid carcinomas [70]. Some studies has evidenced that several serum factors mediating inflammatory processes, angiogenesis, and tumor growth correlate with thyroid cancer development and progression. Benign and malignant thyroid disease often shows inflammatory dysregulation, which may be reflected in distinct serum cytokine profiles [71]. In 2008, Linkov et al. have evaluated by multiplex bead-based immunoassays the concentrations of 19 cytokines, chemokines, and growth factors on sera from patients with cancer, patients with benign nodular thyroid disease, and healthy subjects. They demonstrated that (i) EGF, HGF, IL-5, IL-8, and RANTES had lower levels in subjects with thyroid disease compared to normal controls and (ii) IL-8, HGF, IL-12, and MIG achieved noteworthy discrimination between benign and malignant groups [49]. In recent years it was demonstrated that malignant and benign thyroid conditions are associated with altered expression levels of interleukins, supporting the association between thyroid disease and underlying inflammatory processes. In particular, significantly higher levels of IL-6, IL-7, IL-10, and IL-13, as well as significantly lower levels of IL-8, were observed in patients with benign and malignant thyroid disease compared to controls [50].

## 13. “Cytokinome” and Prostate Cancer

Prostate cancer (Pca) is the most frequent cancer occurring in males, and it has been shown that its pathogenesis involves genetic as well as environmental factors [72]. Recent data suggest that inflammation may play a role in the development of prostate cancer. It has been reported that proinflammatory cytokines are capable of promoting proliferation, invasion, and angiogenesis of prostate cancer [73]. Zhang et al. (2010) have assessed a cytokines profile multiplex array in serum of patients with prostate cancer and benign prostatic hyperplasia, identifying 19 differentially expressed cytokines. Some cytokines, including IL-3, IL-6, and IL-16, ended up being upregulated in prostate cancer patients and others, including Fas/TNFRSF6, TRALR-3, and IGFBP-6, markedly downregulated [74]. On the other hand, in a study of the pathophysiology of cachexia in patients with prostate cancer, a serum cytokinome profile of 10 biomarkers (IL-1*β*, IL-2, IL-4, IL-5, IL-6, IL-8, IL-10, IL-12, IFN-*γ*, and TNF-*α*) was evaluated. From this profile, when compared to that of the control group, it appears that serum levels of all cytokines were significantly higher in the cachexia group, and six cytokines (IL-1*β*, IL-2, IL-8, IL-12, TNF-*α*, and IFN-*γ*) were significantly higher in the group with advanced Pca without cachexia. However, in the group with organ-confined Pca, only IL-1*β* and IL-12 levels were significantly higher compared to the control group [51]. Therefore, the cytokinome profile can be useful to identify new markers of disease progression in presence or absence of other comorbidities.

## 14. Cytokinome Perspectives

One of the great problems in the subclinical chronic inflammation diseases, including cancers, is discriminating which cytokines address the pathogenesis of the various diseases. This is a complex task because immune cells can release many different pathogenic cytokines, which can originate one specific disease or even many and, to complicate matters, inflammatory pathways are highly redundant [75]. This makes it very difficult to identify which specific cytokine hitting to fight a chronic disease. Often, clinically, a proinflammatory cytokine is index of disease, but not of what specific disease; it is at most a confirmation index in the presence of a recognized disease. From this derives the need to identify the whole pattern of cytokines involved in a specific inflammatory disorder, possibly together with the knowledge of the secreting immune cells, because only in this way we can identify key cells as well as the appropriate cytokines for a given inflammatory disorder. In this way we will be able to detect which cytokines might be suitable targets for fighting successfully each disease. On this issue, Schett et al. (2013) [76] share our same view [18], claiming that “human trials targeting different cytokines suggest the existence of a hierarchical framework of cytokines.” This is an important observation leading to differentiation of some homogeneous sets of specific cytokines to classify in different groups the chronic inflammatory disorders which share similar pathogenic pathways in the context of resident tissue cell lineages [77].

Unfortunately, the functional redundancy of the pathways in which the cytokines are involved, also due to the structural pleiotropy of many cytokines [78], made it difficult to specifically target the key cytokines, even with antibodies [78]. This means that many prevalent chronic diseases, believed to be pathophysiologically similar and, therefore, treatable inhibiting the same target cytokine, have shown surprising failures when similarly treated [75]. From this point of view, in our opinion, one of the best available methodological approaches for this purpose is the cytokinome determination [18, 19, 39, 48], that is, the detection of the whole set of cytokines present in a biological fluid in a certain moment. Indeed, classical single cytokine assays fail to capture the physiological dynamics of the whole set of cytokines, due to their limited sensibility of response for the numerous less represented cytokines of which often we still do not have a specific assay [18, 19, 39, 48]. A valid approach to this aim is based on multiplexing measurements where the presence of less abundant cytokines can be expressed as internal ratio [18, 19, 39, 48].

In conclusion, the cytokinome approach by multiplexing measurements, although time dependent, can be very useful to identify cytokine clusters to be specifically hit, for example, by antibodies or other compounds. This methodological approach opens to a new strategy to combat what is the shared basis of almost all the major diseases of modern man, which is the subclinical chronic inflammation, thus, allowing both improving the strategies already in place and doing predictive medicine, especially when we will have a clear taxonomic classification of these diseases with clusters of cytokines linked to them.

## Figures and Tables

**Table 1:** We report for each cancer the list of cytokines of patients with higher or lower levels compared to healthy controls, the type of cohort of patients for which the sera levels of cytokines were evaluated by multiplexed bead-based immunoassays, and related references.

Cancer	Cytokines levels	Type of cohort of patients versus controls	References
Breast cancer	↓TNF-*α* and IL-12	Early stage	[24]
	↑TNF-*α* and IL-1*β*	Advanced stage	[24]
	↑IL-1*β*, IL-6, IL-8, IL-10, and IL-18	Early stage	[26]
	↑IL-2R	Early stage	[26]

Ovarian cancer	↑VEGF, bFGF, PDGF, IL-6, IL-8, IL-1a, IL-1b, MCP-1, G-CSF, M-CSF, and TNF-*α*	Early stage	[31]
	↑angiogenin, angiopoietin-2, GRO, ICAM-1, IL-6, IL-6R, IL-8, IL-10, leptin, MCP-1, MIF NAP-2, osteprotegerin (OPG), RANTES, TIMP-2, and UPAR	Ascites	[32]

Lung cancer	↑sTNFRII, IL-7, TGF-*α*, CXCL5, CXCL9, CXCL13, CCL17, and CCL22	Cancer without other comorbidities	[33]
	↑CCL15	Early stage	[34]
	↑IL-6 and IL-8	Non-small-cell lung cancer	[35]
	↑IL-6, IL-8, IL-12, IL-17, and IFN-*γ* ↓IL-29 and IL-33	Non-small-cell lung cancer	[36]

Colon cancer (CRC)	↑IL-6, IL-7, IL-8, and PDGF-*ββ* ↓MCP-1	T3 or T4 rectal tumors without preoperative radiotherapy or chemoradiotherapy	[37]
	↑IL-1ra, IL-6, IL-8	Advanced stage	[37]
	↑FGF-2, TGF-*α*, Flt-3L, GM-CSF, INF-*α*2, GRO, IL-10, MCP-3, MDC, sIL-2Ra, IL-2, IL-7, IL-8, MCP-1, MIP-1*β*, TNF-*α*, and VEGF,	Metastatic CRC	[38]

Liver cancer (HCC)	↑HGF, IL-2R, s-IL-6Ra, IL-18, leptin, and sVEGFR-1		
	sVEGFR-2, glucagon, *β*-NGF, CXCL1, CXCL9, CXCL12, IL-16, PECAM-1, IFN-*α*, and Prolactin	HCV-related HCC	[39]

Melanoma	↑IL-1a, IL-1b, IL-6, IL-8, IL-12p40, IL-13, G-CSF, MCP-1, MIP-1*α*, MIP-1 *β*, IFN-*α*, TNF-*α*, EGF, VEGF, and TNF-RII	Patients treated with interferon-alpha2*β*	[40]

Gastric cancer	↑IL-6 and IL-8	Early stage	[41]
	↑IL-1*β*, IL-2, IL-4, IL-6, IL-8, IL-10, TNF-*α*, and IFN-*γ*	Distal gastric cancer	[42]
	↑IL-8, eotaxin, HGF, IP-10, MIP-1*β*, VEGF, Gro-a, IL-2R, IL-18, M-CSF, MIF, and MIG	Advanced stage	Our group

Pancreatic cancer	↑IL-10 and TGF-*β*	Primary pancreatic duct adenocarcinomas	[43]
	↑IL-5, IL-6, and IL-10	Stages II-III-IV	[44]
	↑IL-6, IL-8, IL-10, and TNF-*α* ↓IL-23	Newly diagnosed pancreatic adenocarcinoma	[45]

Renal cell carcinoma (RCC)	↑IL-6, IL-10, and VEGF	Metastatic RCC	[46]
	↑IL-5, IL-12, IL-6, and VEGFA	Metastatic RCC	[47]
	↑IL-6, IL-8, IL-10, G-CSF, CXCL10, CXCL11, HGF, and VEGF	RCC after undergoing nephrectomy	[48]

Thyroid cancer	↓EGF, HGF, IL-5, IL-8, and RANTES	Benign and malignant	[49]
	↑IL-6, IL-7, IL-10, and IL-13 ↓IL-8	Benign and malignant	[50]

Prostate cancer (Pca)	↑IL-1*β*, IL-2, IL-4, IL-5, IL-6, IL-8, IL-10, IL-12, IFN-*γ*, and TNF-*α*	Pca with cachexia	[51]
	↑IL-1*β*, IL-2, IL-8, IL-12, TNF-*α*, and IFN-*γ*	Pca without cachexia	[51]
	↑IL-1*β* and IL-12	organ-confined Pca	[51]
